# Assessment of Macro-Level Socioeconomic Factors That Impact Waterborne Diseases: The Case of Jordan

**DOI:** 10.3390/ijerph13121181

**Published:** 2016-11-25

**Authors:** John M. Polimeni, Ahmad Almalki, Raluca I. Iorgulescu, Lucian-Liviu Albu, Wendy M. Parker, Ray Chandrasekara

**Affiliations:** 1 Department of Pharmacy Practice, Albany College of Pharmacy and Health Sciences, 106 New Scotland Avenue, Albany, NY 12208, USA; 2 Department of Obstetrics and Gynecology, King Saud University, King Khalid Road, Riyadh 12372, Saudi Arabia; ahmad.almalki84@gmail.com; 3 Institute for Economic Forecasting-NIER, Romanian Academy, Bucharest 050711, Romania; raluca_i@lycos.com (R.I.I.); albul@ipe.ro (L.-L.A.); 4 Department of Basic & Clinical Sciences, Albany College of Pharmacy and Health Sciences, 106 New Scotland Avenue, Albany, NY 12208, USA; wendy.parker@acphs.edu; 5 Department of Humanities and Communication, Albany College of Pharmacy and Health Sciences, 106 New Scotland Avenue, Albany, NY 12208, USA; ray.chandrasekara@acphs.edu

**Keywords:** water scarcity, waterborne diseases, water pollution, ecological economics, economic growth

## Abstract

The Hashemite Kingdom of Jordan is an example of a country that suffers from high water scarcity. Additionally, due to the economic drivers in the country, such as phosphate and potash extraction and pharmaceutical production, the little fresh water that remains is generally polluted. The infrastructure, often antiquated in urban areas and non-existent in rural areas, also contributes to poor water conditions and to the spread of waterborne diseases. This paper examines the socioeconomic factors that contribute to diarrhea and hepatitis A on a macro level in Jordan and discusses the public-policies that government officials could use to abate those problems. Ordinary least squares time series models are used to understand the macro-level variables that impact the incidence of these diseases in Jordan. Public health expenditure has a significant impact on reducing their incidence. Furthermore, investment in sanitation facilities in rural regions is likely to reduce the number of cases of hepatitis A. Perhaps the most surprising outcome is that importation of goods and services likely results in a decrease in cases of hepatitis A. However, income has little impact on the incidence of diarrhea and hepatitis A.

## 1. Introduction

Water, the vital element for social, environmental, and economic systems, is often not accessible for approximately 11% of the world’s population. There are approximately 780 million people that do not have access to safe drinking water and nearly 2.5 billion people who lack access to proper sanitation and are forced to live in unhealthy communities [1]. Not providing an adequate supply of clean, safe water to a population (due to, for example, the practice of reducing pressure in the urban water system, failure or inability to disinfect the water supply, and leaks in the pipelines), could become, under certain geographical conditions, costlier for a country in health care terms than improving the water infrastructure.

Two global health problems are waterborne diarrhea and hepatitis A. Diarrhea is an infectious disease that accounts for 3.2% of all deaths worldwide and is the third highest cause of mortality, killing every year more than 1.8 million people, mainly children since they are still developing their immune systems and are very vulnerable to environmental hazards and toxins [2,3,4]. Hepatitis A is typically transmitted through food or water and lack of sanitation. This virus infects approximately 212 million people every year, leading to more than 100,000 deaths per year [5]. The majority of cases of diarrhea and hepatitis A in developing, water-scarce countries, such as Jordan, are the result of contaminated water and food sources [6] and their incidence is strongly correlated with various socioeconomic factors [7]. 

For the purposes of this paper, the Falkenmark water stress index [8] is used. The level of water scarcity of a country is defined in terms of total renewable water available annually to its population: (1) “water stress” below 1700 cubic meters per person per year and above 1000 cubic meters; (2) “water scarcity” below 1000 cubic meters and above 500 cubic meters; and (3) “absolute water scarcity” below 500 cubic meters. A country can also be water scarce if annual withdrawal is between 20% and 40% of the annual national supply and it is considered to be in severe water scarcity conditions if the annual withdrawal is more than 40%.

According to the World Resources Institute, the Middle East has the highest density of countries with high or extremely high water stress [9] and Jordan is among those with the greatest water scarcity: more than 90% of the country receives less than 200 millimeters of rain per year and 70% of the country receives less than 100 millimeters of rain per year [10]. Before the worsening of the Syrian war refugee crisis in 2010, the per capita annual share of water was 147 cubic meters and renewable water sources less than 130 cubic meters, significantly below the benchmark of 500 cubic meters per person per year. By 2025, the supply of water is projected to be less than 90 cubic meters per person per year, putting Jordan in an absolute water scarcity situation [11]. For comparison, in 2013, an average US citizen consumed slightly more than 8900 cubic meters of fresh water per year while a Jordanian consumed only 105 cubic meters [12]. 

The majority of deaths from diarrhea and hepatitis A occur in low and middle income countries (of which Jordan, with absolute water scarcity, is an excellent case study). Developing appropriate policies to reduce their impact requires a complex approach with respect to social structures and economic systems. The literature on diarrhea in Jordan finds that the source of infection was linked to contaminated water not boiled used for milk preparation for children younger than two [13]; to untreated rainwater stored in a well and in proximity to animals [14]; to environmental factors such as water contamination which can cause Shigella [15] or rotavirus [16] infections; to the lack of water, breastfeeding, and household structure [17]; to low socio-economic status, low levels of education, use of non-chlorinated well or tank water, and poor hygiene [18]. Besides identifying the specific parasites and causes of the outbreaks—which was the focus of the literature on diarrhea and hepatitis A [19] in Jordan—it is extremely important to understand how and which macro-level socioeconomic factors impact the incidence of those diseases and, consequently, life expectancy.

For water scarce countries, perhaps more than for other countries, macro-level socioeconomic factors (e.g., regional inequality; the standard of living; the unbalanced split rural/urban population; the level of imports of goods and services which would reduce the amount of water consumed for domestic production; the level of access to health care) contribute to an increased risk for waterborne illness and death. Even if these factors are extremely important for assessing and mitigating the impact of waterborne diarrhea and hepatitis A, only in the past decade or so have efforts been made to better understand their role [20]. For example, among other researchers, Yongsi and Ntetu [21] in Cameroon, and Pande et al. [22] in Benin, found that the standard of living has a positive impact on diarrhea, lowering the number of cases of diarrhea. Furthermore, research done in Brazil has found hepatitis A seroprevalence to be higher in low-income populations [23,24,25]. This paper fills a gap in the literature on the macro-level relationships for waterborne diarrhea and hepatitis A in Jordan since its approach is different from previous analyses that are local or site-specific. The results of this research provide Jordanian policymakers with quantitative data useful in developing a health care strategy while also helping to improve life expectancy. We are not aware of any previous study.

## 2. “Debriefing” on Absolute Water Scarcity and Its Health Impact in Jordan

One of the factors contributing to the absolute water scarcity crisis in Jordan is the rapid increase in population, from 470,000 in the early 1950s to more than 6 million today and still growing [26]. Also increasing urbanization, rising living standards, and an influx of refugees from Syria (more on this issue in the discussion section) have generated a higher demand for water. Jordan’s freshwater supply, including surface (65%) and groundwater (35%), is roughly 780 to 850 million cubic meters per year [27]. There is projected to be an increase in total water supply from 1029 million cubic meters in 2005 to 1296 million cubic meters in 2020. Overall the amount of available water has increased, but the consumption rate for municipal use increased at a higher rate, from 20% in 1992 to 29% in 2000 [28] and by 2020 it is estimated that, under normal circumstances, Jordan could have a water deficit of 319 million cubic meters [29]. 

The major surface water sources are the Jordan River (shared with Lebanon, Syria, Palestine, and Israel) and its tributaries, the Yarmouk and Zarqa rivers which are both shared with other countries; the management of the water supply from those sources is not completely under the Jordanian government. To make things worse, the extraction rate from groundwater sources is estimated to be 50% above the safe level and has led to a significant reduction in groundwater quantity and quality. Rainfall is the only water source which refills those aquifers and more than 90% of it is thought to be lost immediately due to evaporation [26]. In 2000, 94% of the water used for industry came from groundwater [30]. As a result, Jordan imports food supplies, mainly wheat and other cereals, as well as vegetable oil, to compensate for the water shortage [29]. Economic activity in Jordan is primarily composed of the service sector and an industry dominated by phosphate and potash extraction and pharmaceutical production. Over the decade 2000–2010, these economic drivers have been effective. This is evidenced by GDP growth, from approximately $8.4 billion to nearly $16 billion in constant 2000 dollars [31]. Similarly, Gross National Income (GNI) also nearly doubled over the same time period. In contrast, industrial pollution is very high, likely contributing to the incidence of diarrhea and hepatitis A through contaminated water. 

The Jordanian Ministry of Water and Irrigation is responsible for monitoring and managing the freshwater supply and wastewater system. In 1997, the Jordanian government established, under the supervision of the World Bank, a policy for the management of water resources [32]. As a consequence, a private water sector was established in 1999 in Amman, and a National Water Master Plan (NWMP) was established in 2004. 

The NWMP’s main objective is it to reach an internationally approved minimum standard of 100 liters per person per day by 2020. According to Nortcliff et al. [30], reaching this level is a challenge since between 1996 and 2001 the supply of water decreased from 103 liters per person per day to 86. Much of this decline was due to infrastructure defects, which accounted for 30% of the loss. In the year 2000, an estimated 50% of water supplied for municipal usage was considered unaccounted for, and within the Jordan Valley 35% of the irrigation water was also unaccounted for. 

Given the geography and the economic and water stress situation, in Jordan, the cases of diarrhea far outnumber the hepatitis A cases. From the years 2000 to 2010, according to official Jordanian Ministry of Health data [33], the average number of cases of diarrhea per year was 117,894 as compared to 502 cases of hepatitis A. 

**Figure 1:**
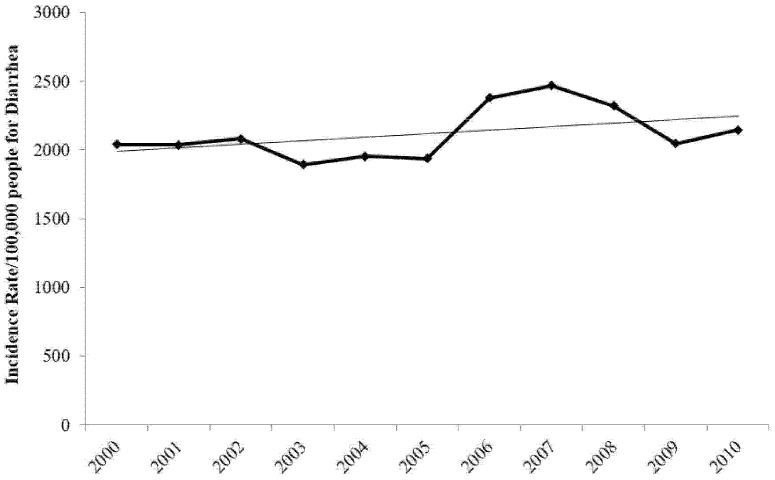
Incidence rate per 100,000 people for diarrhea in Jordan (2000–2010). Source: Jordanian Ministry of Health data [33].

During the study timeframe, the incidence rate of diarrhea (Figure 1) has increased as the trend-line shows. The increase might be partially explained by the large number of organisms which can cause diarrhea, of which some might be endemic with no preventive methods to counteract them. 

**Figure 2:**
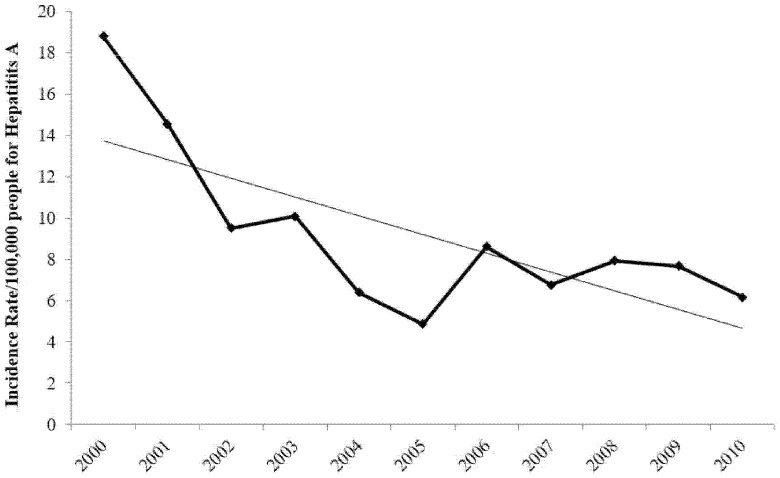
Incidence rate per 100,000 people for hepatitis A in Jordan (2000–2010). Source: Jordanian Ministry of Health data [33].

On the other hand, Jordan has experienced a major decrease in the incidence rate of hepatitis A, as indicated by the trend-line in Figure 2. This decrease is consistent with public policy in Jordan curtailing this disease through children vaccination.

**Figure 3:**
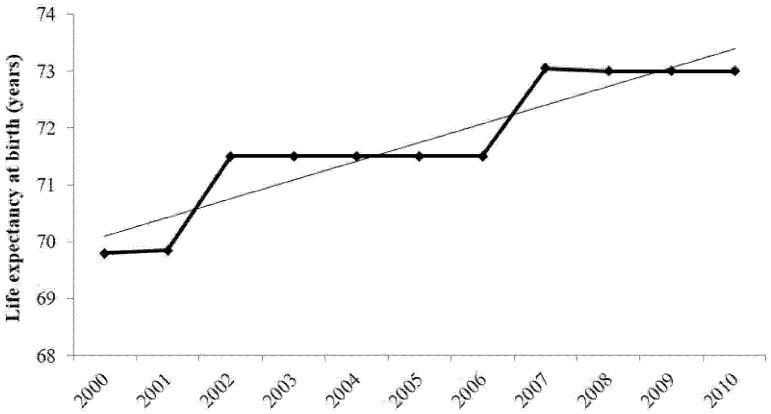
Life expectancy in Jordan (2000–2010). Source: Jordanian Ministry of Health data [33].

As a result, life expectancy during the study period increased, as shown by the trend line in Figure 3, which points to the potential success of Jordanian government policies. 

Before the Syrian war tore apart the region, despite the efforts of the Jordanian government there still was a high level of incidence of diarrhea and hepatitis A. Understanding the macro-level socioeconomic variables that impact the number of cases of diarrhea and hepatitis A and, implicitly, life expectancy could enable the Jordanian government to further target mitigation policies for these diseases.

## 3. Materials and Methods

### 3.1. Data

In 1982, after Jordan established the diarrheal disease control program, it became mandatory to report, weekly and monthly, any cases of diarrhea to the Ministry of Health. In Jordan, there are 21 health reporting regions and many healthcare providers which are responsible for notifying officials about communicable diseases, including the Ministry of Health, university hospitals, the Royal Medical Services, private sector providers, and the United Nations Relief and Work Agency (UNRWA). UNRWA also collects data from all refugees in Jordan and reports regularly to the Department of Communicable Diseases (DCD, established in 1993) on a weekly and monthly basis [33]. Data, covering the decade 2000–2010 (before the Syrian war became an external shock for Jordan), were obtained from the Jordanian Ministry of Health Department of Communicable Diseases for incidence of diarrhea and hepatitis A and from the World Bank for macro-level socioeconomic variables. 

### 3.2. Variables

Typically there is a positive relationship between health, standard of living, and income [34]. Bosworth and Burke [35] did an extensive study illustrating the positive relationship between income and life expectancy. Novignon et al. [36] and the Organisation for Economic Co-operation and Development (OECD) [37] both found a positive relationship between public and private health expenditure, health expenditure per capita, and life expectancy. Consequently, healthcare expenditure variables are important to the analysis presented in this paper since one can also extrapolate that these expenditures have a negative relationship with waterborne diseases such as diarrhea and hepatitis A.

The total population of Jordan increased from approximately 4.8 million to nearly 6.2 million people from 2000 to 2010. Since urban population increased by nearly the same number (3.8 million to 5.2 million people) while rural population increased by slightly more than 100,000 people [31], the split rural/urban population is a good proxy for urbanization and, implicitly, better sanitation and water services. 

Different variables were considered but were not significant; subsequently a combination of fifteen independent variables in ten models with three different dependent variables (*cases of diarrhea*, *cases of hepatitis A*, and *life expectancy*) were chosen for this analysis (Table 1 for a list of these variables and their expected sign). In addition, the *incidence rate of hepatitis A* as a dependent variable was examined using one model. Models of the *incidence rate of diarrhea* were tested but none resulted in meaningful findings or had good performance.

### 3.3. Model

Many studies on the incidence of diarrhea and hepatitis A use a mathematical model to predict mortality or their spread. Less common, however, is the use of linear regression, especially using macro-level socioeconomic factors, to understand the variables that impact the incidence of those diseases. Pande et al. [22] used a mixed effect logit model that applied spatial data at the household level to predict the prevalence of diarrhea in the Oueme River Basin. Ghias and Pervaiz [38] used a multiple linear logistic regression model to find the factors associated with hepatitis A. Ardkaew and Tongkumchum [3] used linear regression models that included the district, year, and season fitted to the log-transformed incidence of diarrhea with generalized estimating equations to account for spatial correlation. Bekti and Sutikno [39] continued this line of research comparing ordinary least square regression and a spatial Durbin model to find that the spatial Durbin model was able to identify spatial factors that influence incidence of diarrhea that ordinary least square regression could not; otherwise, the two models provided similar results. 

Since the data used in this analysis does not contain a spatial component, ordinary least squares (OLS) with time series data is used for the analyses of the three dependent variables in ten models. Furthermore, since socioeconomic macro-level data are not used very often in the literature as predictor variables, the approach here is distinctive. All the models take the form shown in Equation (1).

(1) $$Y_{t}=\beta_{1}+\beta_{2}X_{t}+e_{t}$$

where:

*Y_t_* = dependent variable over time (life expectancy, incidence of diarrhea, incidence of hepatitis A)
*β*_1_ = intercept
*β*_2_ = slope and coefficient of independent variables
*X_t_* = matrix of independent variables
*e_t_* = error term

All models were checked for multicollinearity, autocorrelation and heteroskedasticity and the results presented below are those after the tests and any needed corrections. Additionally, all the variables were checked for stationarity and, as a result, variables were differenced to become stationary.

## 4. Results and Discussion

### 4.1. Diarrhea

Three models (Di) are used to examine the number of cases of diarrhea in Jordan from 2000 to 2010 (Table 2). 

Model D1 explores how rural population growth, urban population growth, GDP per capita, and health expenditure per capita explain cases of diarrhea in Jordan. All of the variables have the expected signs on their coefficients. Both rural population growth and urban population growth have the largest magnitudes, indicating a great divergence in the sanitation facilities between rural and urban regions. However, the model does not fit the data points exceptionally well, with an R-squared of 69.7%. 

Therefore, model D2 replaced GDP per capita with GDP as a measure of affluence and replaced health expenditure per capita with out-of-pocket health expenditure to examine if individual wealth and direct health costs have an impact on diarrhea. Urban population growth and rural population growth remained in the model. GDP has a positive coefficient, suggesting that Jordan’s production could be polluting the water supply. However, the impact of GDP is negligible since the magnitude of the coefficient is essentially zero. As expected, urban population growth has a negative coefficient and rural population growth has a positive coefficient. As explained in the previous section, this result is likely due to better quality drinking water and sanitation infrastructure in urban areas as opposed to rural areas. Rural regions in developing countries are unlikely to have the appropriate water supply and sanitation infrastructure to provide safe water. Out-of-pocket health expenditure has a positive coefficient since due to the greater the incidence of diarrhea, people will pay more for services and products to treat the illness. The results of the model, given the magnitude of the coefficients, suggest that encouraging urbanization and investing in infrastructure in rural areas is a way for the Jordanian government to reduce the number of cases of diarrhea. The R-squared of this model was nearly 84.7% and the adjusted R-squared 72% indicating that this is a much better fit of the data than in model D1. 

With the relatively good results of model D2 and the strength of the magnitude of out-of-pocket health expenditure, model D3 builds on these findings to examine the impact of public health expenditure and private health expenditure. The performance of this model improved slightly with an R-squared of 85.4% and an adjusted R-squared of 73.7%. Both public and private health expenditure are negative and have large magnitudes. However, the magnitudes of the coefficients for rural population growth and urban population growth are greater, indicating their higher importance. 

### 4.2. Hepatitis A

Although the disease is contracted in similar ways, hepatitis A often impacts a different subsection of people. As a result, three models (HAi) are used to explain the incidence of hepatitis A in Jordan from 2000 to 2010 (Table 3). 

The first model (HA1) uses imports of goods and services, final consumption expenditure, out of pocket health expenditure, and total population. All of the variables have signs on their coefficients that are expected. The variable that has the strongest relationship with hepatitis A is out-of-pocket health expenditure, which is significant at the 90% level. This result is intuitive as people will have to spend more of their money when they get sick. The model explained cases of hepatitis A well with a 71% R-squared and an adjusted R-squared of 48%.

Model HA2 attempts to improve these results using the percentage of the population that is female, rural and urban population growth, and the percentage of the rural population with access to sanitation facilities. This model performed slightly better than the first model with an R-squared of 73% and an adjusted R-squared of 51%. The signs of the coefficients for all the variables are as expected. The percentage of rural population with access to sanitation facilities has the greatest impact of the variables used in this model and combined with the result for rural population growth strongly suggests that investment in rural sanitation services can significantly reduce the incidence of hepatitis A. 

Model HA3 attempts to build upon the results of the second model by using the percentage of the rural population with access to sanitation facilities, imports of goods and services, household consumption expenditure, and public and private health expenditure. This model explained the data with the best of the three, with an R-squared of 80% and an adjusted R-squared of 56%. The percentage of the rural population with access to sanitation facilities has the greatest impact of all the variables, although both public and private health expenditure have a large influence as well. The results suggest that government investment in rural sanitation services and healthcare can have a significant impact on reducing hepatitis A.

In addition to the above, one model (Table 4) examines how the incidence rate of hepatitis A (IRHA) is impacted by private health expenditure, imports of goods and services, household consumption expenditure, the percent of the rural population with access to sanitation facilities, and public health expenditure.

All the variables, with the exception of household consumption expenditures, have negative coefficients. The percentage of the rural population with access to sanitation facilities has the greatest impact on reducing the incidence rate of hepatitis A, although private health expenditures and imports of goods and services also have negative coefficients and substantial magnitudes. The model explained the incidence rate of hepatitis A well, with an 81.3% R-squared value. These results suggest that government investment in rural sanitation services and private health expenditures will decrease the incidence rate of hepatitis A. Furthermore, the negative coefficient for imports of goods and services indicates that the production of goods and services in Jordan cause environmental damage that contributes to the incidence of hepatitis A. 

### 4.3. Life Expectancy

The final set of models (LEi) examines which macro-level variables impact life expectancy in Jordan. Life expectancy is tested as an independent variable to examine whether the number of cases of diarrhea or of cases of hepatitis A impacts it. In the research undertaken for this paper, only the number of cases of diarrhea was found to be significant in impacting life expectancy. Four models are used to examine life expectancy from a macro-level perspective (Table 5).

Model LE1 uses the percentage of the population that is female, GDP per capita, and private health expenditure. The model performed well with an R-squared of 89.9% and an adjusted R-square of 84.8%. All the variables were significant at the 98% level or higher. However, some of the variables, such as the percentage of the population that is female and GDP per capita, have signs on the coefficients that are not expected. One possible explanation for the unexpected sign for the percentage of the population that is female is that less than 50% of the population is female, indicating that there might have been a preference for male babies, particularly in the earlier time period of this study [40]. Another possible explanation is that rural women are more exposed to viruses and contaminants working in the fields and running their households. The reason for the negative sign on the coefficient for GDP per capita might be related to the reported serious and increasing regional economic inequality [41]. Theoretically, an increasing GDP per capita corresponds with better life expectancy.

Model LE2 attempts to eliminate some of these issues by using as new independent variables cases of diarrhea, out-of-pocket health expenditure, total population, and gross national income. This model yielded results similar to the first model with an R-squared of 91% but an adjusted R-squared of 83.7%. All the variables are significant at the 93% level or higher. Out-of-pocket health expenditure and total population have signs on the coefficients as expected. However, cases of diarrhea and gross national income both have coefficients that are opposite of expected. With regard to cases of diarrhea, although it has a positive coefficient, the magnitude is zero. One potential explanation for this result is that the longer people live the greater the likelihood that someone will get diarrhea which would not be life threatening. On the other hand, gross national income has a negative coefficient when a positive one was expected, possibly in relation to regional economic inequality [41]. None of the coefficients for the variables have a large magnitude; however, out-of-pocket health expenditure has the greatest impact of those variables used in this model.

Model LE3 attempts to improve upon the results of the second model. This model uses the percentage of the population that is female, health expenditure per capita, the percentage of the urban population with access to sanitation facilities, and GDP to explain life expectancy. Results of this model are improved over the previous two, with an R-squared of 92.2%, an adjusted R-squared of 86%, and all the variables are significant at the 90% level or higher. All of the variables have coefficients as expected with the exception of the percentage of the population that is female. As with the previous model, the reason for the unexpected coefficient sign for the percentage of the population that is female is similar. Interestingly enough, the variables with the greatest magnitudes are the percentage of the population that is female and the percentage of the urban population with access to sanitation services. The urban population access to sanitation facilities shows the importance of sanitation services to the Jordanian population and that if the Jordanian government seeks to improve the health and life expectancy of its people then investment in sanitation infrastructure would be an efficient method of doing so. 

The fourth and final model (LE4) uses the findings of the previous three models and incorporates the percentage of the population that is female, health expenditure per capita, the percentage of the urban population with access to sanitation facilities, and GDP per capita as independent variables to improve upon the results. There are improvements with this model, with an R-squared of 93.4% and an adjusted R-squared of 88%. Additionally, all the variables are significant at the 90% level or higher. However, only health expenditure per capita and the percentage of the urban population with access to sanitation facilities have the expected signs of the coefficients. As with the previous models, the reason for the negative coefficient for the percentage of the population that is female is likely to be because less than 50% of the population is female, while the reason for the sign on GDP per capita might relate to increasing regional economic inequality [41]. As in the third model, both the percentage of the population that is female and the percentage of the urban population with access to sanitation facilities have the greatest impact on life expectancy. 

This finding suggests that the Jordanian government can have a fairly substantial positive impact on life expectancy by investing in infrastructure that improves the sanitation capabilities of the population and through measures aimed at decreasing regional inequality. 

## 5. Conclusions

Usually, in studies of diarrhea, hepatitis A, and life expectancy in Jordan (outlined previously in this paper), surveys are conducted in various towns and villages in the country to quantify the incidence of these diseases and to identify the specific organisms causing the problem. Given that these diseases are typically caused by contaminated water supply or inadequate sanitation services, macro-level socioeconomic determinants of health must also be included in an analysis for policy makers because of the complexity and interconnectedness of economic and environmental systems.

In this paper, the study was restricted to the decade 2000–2010 in order to avoid the issues related to the Syrian refugee crisis. Since the influx of more than 7 million Syrian refugees, Jordan is now considered the third most water insecure country in the world [26]. In fact, since 1948 the Middle East crises have exacerbated the water crisis in Jordan. As these crises continue and more arise, the availability of water in Jordan becomes a direr problem. Using official data on diarrhea and hepatitis A from the Jordanian Ministry of Health, Department of Communicable Diseases, this study examined for the first time to the best of our knowledge, Jordanian macro-level socioeconomic factors that may influence the environmental conditions that cause diarrhea, hepatitis A, and life expectancy. 

The findings provide insight into which public policies could have the greatest impact in counteracting the public health problems of diarrhea and hepatitis A in Jordan. Not surprisingly, public health expenditure has a significant impact in reducing the incidence of diarrhea and hepatitis A. As a result, if the Jordanian government seeks to reduce these diseases, public health care spending could be an effective way of doing so. Given the magnitude of the difference between access to sanitation services in rural and urban areas and the impact of rural and urban population growth, investment in sanitation facilities in rural regions will likely lead to great reductions in hepatitis A. Perhaps the most surprising outcome is that imports of goods and services likely result in a decrease in cases of hepatitis A. This finding suggests that domestic production is polluting the environment and contributing to the incidence of hepatitis A. Furthermore, as stated previously, food is a major import for Jordan, suggesting that perhaps this food is produced in better sanitary conditions than the food produced in Jordan. One other surprising result is the lack of impact of income variables on the incidence of diarrhea and hepatitis A.

In terms of life expectancy, the findings were, for the most part, as expected. Private health expenditure has a positive relationship with life expectancy, indicating that those individuals that actively seek healthcare live longer. While this result is not surprising, the finding can be used in a campaign by the Jordanian government to persuade people that actively participating in their healthcare will lead to a longer life. Furthermore, the percentage of the urban population with access to sanitation facilities has a large positive impact on life expectancy. Combined with the results from the models for incidence of diarrhea and hepatitis A, this finding strongly suggests that the Jordanian government should invest in sanitation facilities in the country. The main surprising result for the models of life expectancy is that the percentage of the population that is female has a large negative effect. This result is particularly unexpected given that women have a longer life expectancy then men in Jordan. However, this finding could be because women, especially in rural communities, are more exposed to viruses and contaminants by working in the fields and in their duties running their households. As in the models of diarrhea and hepatitis A, the income variables used in the life expectancy models have little impact but they indicate the need to address regional economic inequality.

Both diseases are water related and, as such, a strategy to counteract these ailments would likely be in the form of better infrastructure for water supply and sanitation. Water shortages in Jordan will only worsen as climate change impacts the country. Since Jordan has a small economy, with a GDP per capita of approximately $6,100 and substantial budgetary and balance of payments imbalances, investments in infrastructure, such as those suggested in this paper, will be difficult. Contributing to the problem is the influx of refugees from countries with political instability in the region which puts additional stress on Jordan’s municipal services. However, given the strong connection between the public health of a population and economic growth, these investments are likely to result in greater gains, such as improvements in GDP (through improved health of workers) and a reduction in public health expenditure, which will improve the Jordanian economy. 

A major investment in technologies and infrastructure is needed to positively impact the intermittency, quality, and availability of the water supply. Given the humanitarian crisis with the influx of Syrian refugees into Jordan, the situation will only get worse. According to the United Nations [26], in informal settlements, the per capita consumption of water is estimated to be between 25 and 50 L per day. Furthermore, the country developed a national water strategy for the years 2008–2022 based upon a population of approximately 7.8 million people; the flood of refugees into the country has lifted the current population to nearly 8 million people already. For comparison, to ensure good personal hygiene daily water consumption is estimated to be a minimum of 50 to 100 L, otherwise people are vulnerable to diarrhea and hepatitis A, particularly children and pregnant women [26].

The research presented in this paper is important because, to our knowledge, for the first time there is quantitative data using official statistics on cases of diarrhea and hepatitis A that show what macro-level socioeconomic factors have an impact on reducing the incidence of these diseases and improving life expectancy which provide policymakers important information on how best to proceed to benefit their fellow Jordanians. 

## Figures and Tables

**Table 1:** Description of the variables used.

Measure of	Variable Name	Description	Expected Sign		
			*Cases of Diarrhea*	*Cases of Hepatitis A*	*Life Expectancy*
	Cases of diarrhea				−
	Cases of hepatitis A				
**Standard of living**	GDP		+		+
	GDP per capita		+		+
	Gross National Income (GNI)				+
**Living standard and poverty**	Household final consumption expenditure	Household consumption increases the amount of environmental degradation through improperly disposed waste.		+	
	Imports of goods and services	Imports are likely to reduce the level of domestic environmental degradation from production.		−	
**Health expenditure**	Health expenditure per capita		−	−	+
	Out-of-pocket health expenditure	Percentage of GDP that incorporates any household payment to health practitioners, pharmaceutical supplies, therapeutic products, and all other healthcare goods and services [31]	+	+	−
	Private health expenditure	Percentage of GDP that incorporates all health-related out-of-pocket spending and private insurance costs [31]	−	−	+
	Public health expenditure	Percentage of total health spending on government disbursements for healthcare [31]	−	−	+
**Regional impact**	Percentage of rural population with access to sanitation facilities		−	−	
	Percentage of urban population with access to sanitation facilities				+
**Population**	Total population		+	+	
	Rural population growth		+	+	
	Urban population growth		+	+	
	Percentage of population female			−	+

−: Negative relationship; +: Positive relationship.

**Table 2:** Factors influencing the number of cases of diarrhea in Jordan (years 2000–2010).

Variable	Model D1	Model D2	Model D3
**Constant**	−1746.66	−7127.84	−6792.25
	(3704.60)	(4379.13)	(3620.56)
**Rural Population Growth**	170,581.40 *	146,327.50 ***	186,263.80 ***
	(82,167.66)	(42,639.89)	(49,460.77)
**Urban Population Growth**	−222,340.30 *	−160,105.20 *	−233,076.40 **
	(91,438.25)	(79,550.51)	(67,637.01)
**GDP per Capita**	35.52 *		
	(14.42)		
**Health Expenditure per Capita**	−332.34 **		
	(97.24)		
**Out-of-Pocket Health Expenditure**		2546.85 ***	
		(622.46)	
**GDP**		0.000005 **	
		(0.000002)	
**Public Health Expenditure**			−8512.22 **
			(2698.83)
**Private Health Expenditure**			−36,546.60 **
			(12,114.13)
***R-squared***	0.697	0.847	0.854
***Adjusted R-squared***	0.454	0.724	0.737

Note: ***, **, and * denote statistical significance at the 0.01, 0.05 and 0.10 levels, respectively; standard errors in parentheses. Source: Calculated based on Eviews software.

**Table 3:** Factors influencing the number of cases of hepatitis A in Jordan (years 2000–2010).

Variable	Model HA1	Model HA2	Model HA3
**Constant**	−3563.58 *	1005.06 **	155.13
	(1465.48)	(372.60)	(196.58)
**Percentage of Population Female**		−2402.86 **	
		(766.14)	
**Rural Population Growth**		2507.17 *	
		(1064.51)	
**Urban Population Growth**		−3627.44 *	
		(1785.16)	
**Percent of Rural Population with Access to Sanitation Facilities**		−4539.09 **	−2178.33
		(1782.59)	(1081.93)
**Imports of Goods and Services**	−0.000000233 *		−0.000000283 **
	(0.00000008)		(0.00000008)
**Final Consumption Expenditure**	0.0000003 **		
	(0.0000001)		
**Household Consumption Expenditure**			0.000000392 **
			(0.0000001)
**Out-of-Pocket Health Expenditure**	33.44 *		
	(15.97)		
**Total Population**	0.03 *		
	(0.012)		
**Public Health Expenditure**			−130.72 *
			(58.11)
**Private Health Expenditure**			−498.78 *
			(205.64)
***R-squared***	0.71	0.73	0.80
***Adjusted R-squared***	0.48	0.51	0.56

Note: ** and * denote statistical significance at the 0.05 and 0.10 levels, respectively; standard errors in parentheses. Source: Calculated based on Eviews software.

**Table 4:** Factors influencing the incidence rate of hepatitis A in Jordan (years 2000–2010).

Variable	Model IRHA
**Constant**	2.48 **
	(0.82)
**Private Health Expenditure**	−10.08 *
	(3.89)
**Imports of Goods and Services**	−0.000000005 **
	(0.000000002)
**Household Consumption Expenditure**	0.000000007 **
	(0.000000002)
**Percent of Rural Population with Access to Sanitation Facilities**	−40.63 ***
	(0.92)
**Public Health Expenditure**	−2.50 **
	(21.72)
***R-squared***	0.813
***Adjusted R-squared***	0.581

Note: ***, **, and * denote statistical significance at the 0.01, 0.05 and 0.10 levels, respectively; standard errors in parentheses. Source: Calculated based on Eviews software. IRHA: Incidence of hepatitis A.

**Table 5:** Factors influencing life expectancy in Jordan (2000–2010).

Variable	Model LE1	Model LE2	Model LE3	Model LE4
**Constant**	0.147 ***	0.001	0.159 ***	0.154 ***
	(0.005)	(0.052)	(0.010)	(0.007)
**Percentage of Population Female**	−0.19 ***		−0.25 ***	−0.23 ***
	(0.030)		(0.049)	(0.035)
**Health Expenditure per Capita**			−0.00016 *	−0.00014
			(0.00008)	(0.00007)
**Percent of Urban Population with Access to Sanitation Facilities**			0.25 *	0.23 *
			(0.12)	(0.10)
**GDP per Capita**	−0.00001 **			−0.00004 *
	(0.000004)			(0.00002)
**Cases of Diarrhea**		0.0000004 **		
		(0.0000001)		
**Out-of-Pocket Health Expenditure**		−0.002 *		
		(0.0005)		
**Total Population**		0.0000009 *		
		(0.0000003)		
**Gross National Income**		−0.00002 *		
		(0.000009)		
**Private Health Expenditure**	0.015 ***			
	(0.004)			
**GDP**			0.000000000007 *	
			(0.000000000001)	
***R-squared***	0.899	0.910	0.922	0.934
***Adjusted R-squared***	0.848	0.837	0.860	0.881

Note: ***, **, and * denote statistical significance at the 0.01, 0.05 and 0.10 levels, respectively; standard errors in parentheses. Source: Calculated based on Eviews software.
